# Colloid Preload versus Crystalloid Co-Load in the Setting of Norepinephrine Infusion during Cesarean Section: Time and Type of Administered Fluids Do Not Matter

**DOI:** 10.3390/jcm12041333

**Published:** 2023-02-07

**Authors:** Kassiani Theodoraki, Sofia Hadzilia, Dimitrios Valsamidis, Konstantina Kalopita, Emmanouil Stamatakis

**Affiliations:** 1 Department of Anesthesiology, Aretaieion University Hospital, National and Kapodistrian University of Athens, 11528 Athens, Greece; 2 Department of Anesthesiology, Alexandra General Hospital of Athens, 11528 Athens, Greece

**Keywords:** cesarean section, norepinephrine, hypotension, spinal anesthesia, blood pressure, crystalloid, colloid, preloading, co-loading

## Abstract

**Background and Goal of Study**: Spinal anesthesia for cesarean section is frequently associated with a high incidence of hypotension, which may bring about untoward effects for both the mother and fetus. Recently, norepinephrine has emerged as a promising alternative in maintaining blood pressure in the obstetric setting. Fluid administration is another technique still widely used to prevent maternal hypotension. The optimal fluid strategy to prevent maternal hypotension has not been elucidated yet. It has been recently suggested that the main strategy in the prevention and management of hypotension should be the combination of vasoconstrictive medications and fluid administration. The aim of this randomized study was to compare the incidence of maternal hypotension in parturients receiving either colloid preload or crystalloid co-load in the setting of prophylactic norepinephrine infusion during elective cesarean section under combined spinal–epidural anesthesia. **Materials and Methods**: After ethics committee approval, 102 parturients with full-term singleton pregnancies were randomly allocated to either 6% hydroxyethyl starch 130/0.4 5 mL/kg before the onset of spinal anesthesia (colloid preload group) or Ringer’s lactate solution 10 mL/kg concurrent with the subarachnoid injection (crystalloid co-load group). In both groups, norepinephrine 4 μg/min starting simultaneously with the administration of the subarachnoid solution was also administered. The primary outcome of the study was the incidence of maternal hypotension, defined as systolic arterial pressure (SAP) <80% of baseline. The incidence of severe hypotension (SAP < 80 mmHg), total dose of vasoconstrictive agents administered, as well as the acid–base status and Apgar score of the neonate and any incidence of maternal side effects were also recorded. **Results**: Data analysis was performed on 100 parturients: 51 in the colloid preload group and 49 in the crystalloid co-load group. No significant differences were demonstrated between the colloid preload group and the crystalloid co-load group in the incidence of hypotension (13.7% vs. 16.3%, *p* = 0.933) or the incidence of severe hypotension (0% vs. 4%, *p* = 0.238). The median (range) ephedrine dose was 0 (0–15) mg in the colloid preload group and 0 (0–10) mg in the crystalloid co-load group (*p* = 0.807). The incidence of bradycardia, reactive hypertension, requirement for modification of vasopressor infusion, time to the first occurrence of hypotension, and maternal hemodynamics did not differ between the two groups. There were no significant differences in other maternal side effects or neonatal outcomes between groups. **Conclusions**: The incidence of hypotension with a norepinephrine preventive infusion is low and comparable with both colloid preload and crystalloid co-load. Both fluid-loading techniques are appropriate in women undergoing cesarean delivery. It appears that the optimal regimen for prevention of maternal hypotension is a combined strategy of a prophylactic vasopressor such as norepinephrine and fluids.

## 1. Introduction

Neuraxial techniques have long been established as the anesthetic techniques of choice for cesarean section because with their application, risks inherent in the use of general anesthesia, such as failed intubation, regurgitation, aspiration of gastric contents, and untoward awareness, are avoided [1]. With neuraxial techniques, the mother can remain awake and fully participate in the birthing experience, thus facilitating early bonding with the neonate. Additionally, the benefits of excellent postoperative analgesia with neuraxial blocks cannot be overemphasized.

Spinal anesthesia specifically, due to its quick and predictable onset, has become the most widely used technique for both elective and emergency cesarean sections. Unfortunately, hypotension due to sympathetic blockade may occur in up to 80% of parturients subjected to spinal anesthesia for cesarean section [2]. Hypotension may lead to adverse maternal side effects such as nausea, emesis, or dizziness. Occasionally, severe hypotension may manifest as impaired fetal oxygenation and fetal acidosis of compromised or premature fetuses as a result of uteroplacental hypoperfusion, while, if left untreated, sustained hypotension may culminate in deleterious complications for the parturient, such as loss of consciousness, aspiration, apnea, cardiovascular collapse, or arrest [3].

A continuous phenylephrine infusion has been advocated as one of the standard approaches in the prevention of maternal hypotension. However, the most prominent side effect of phenylephrine is baroreceptor-mediated reflex bradycardia, which can adversely affect maternal cardiac output. This may limit its usefulness in parturients with cardiac comorbidities or in cases with fetal compromise [4]. Recently, norepinephrine has emerged as a promising alternative in managing hypotension in the obstetric setting, and various studies investigating its use either via bolus administration or as a continuous infusion have arisen in the obstetric literature, demonstrating its beneficial effect [5,6,7,8]. Norepinephrine, due to its weak, dose-dependent β-action apart from its strong alpha-agonist activity, is associated with an inferior prevalence of maternal bradycardia and thus with lower propensity to decrease cardiac output [9].

Although the main strategy for vascular tone maintenance in the obstetric anesthesia setting appears to be the administration of a vasoactive agent, another technique widely used to prevent maternal hypotension is fluid administration, which continues to be part of many obstetric anesthesiologists’ armamentarium [10]. In fact, despite the effectiveness of vasoactive medications, a significantly higher incidence of hypotension has been observed when the vasoactive substance is administered alone without the concomitant administration of fluids [11]. Factors that can be manipulated during fluid administration apart from volume and rate of administration are the type of fluids administered (crystalloids versus colloids) and the timing of administration (preloading, that is, administration before the sympathetic blockade induced by spinal anesthesia, versus co-loading, that is, administration concomitantly with the intrathecal administration of the local anesthetic) [12].

Different fluid-loading regimens have been investigated, while no single approach has been embraced as the gold standard. There is a growing body of evidence that preloading of colloids is superior to preloading of crystalloids in the prevention of hypotension, while co-loading of crystalloids appears to be of equal effectiveness to co-loading of colloids [10,13,14]. Regarding the timing of administration, preloading of crystalloids appears to be an ineffective strategy as compared to co-loading of crystalloids [15,16]. When the optimal time of colloid administration is considered, available data are not clearly in favor of co-loading since the comparison between preloading and co-loading of colloids does not show significant differences in the incidence of hypotension, requirements for vasoconstrictive agents, or neonatal outcome [10,14,17].

A comparison scarcely performed is between colloid preloading and crystalloid co-loading, with just one randomized study available in the literature [18]. Therefore, it has been suggested that this comparison should be further investigated. There is a paucity of literature comparing crystalloids and colloids in the presence of a vasoactive infusion. In fact, from our literature search, the comparison between colloid preloading and crystalloid co-loading has never been performed under the prophylactic administration of a vasoactive agent such as norepinephrine.

According to recent network meta analyses, fluid administration should be supplemented by continuous infusion of a vasoactive agent to achieve the most optimal outcome in the prevention and management of maternal hypotension [10,14]. In fact, combination techniques may be more effective than single interventions. We hypothesized that colloid preload will be superior to crystalloid co-load in the setting of a background norepinephrine infusion. Therefore, the aim of the current randomized controlled trial was to compare the incidence of post-spinal-anesthesia-induced hypotension in parturients administered either colloid preload or crystalloid co-load in the setting of prophylactic norepinephrine infusion during elective cesarean section. Additionally, we sought to investigate the effect of either strategy on neonatal outcome, umbilical cord blood gases, as well as any potential maternal side effects encountered.

## 2. Methods

### 2.1. Trial Design, Ethics, and Participants

The protocol of this randomized controlled trial was approved by the Alexandra General Hospital of Athens Ethics Committee (No 302/26-05-2020; date of approval 26 May 2020) and was prospectively registered on clinicaltrials.gov under number NCT04406051 before the first parturient enrolment (principal investigator K.T.; date of registration 28 May 2020). The research design was in accordance with the Consolidated Standards of Reporting Clinical Trials (CONSORT) [19] and took place in compliance with the Helsinki Declaration. Parturients were recruited from June 2020 to June 2021. One hundred and two American Society of Anesthesiologists (ASA)-II-termed uncomplicated singleton gestation parturients scheduled for elective cesarean section were enrolled in the study after being assessed for eligibility during the preoperative visit and after providing written informed consent. Exclusion criteria were age <18 years old, body mass index (BMI) >40 kg m^−2^, body weight less than 50 kg or more than 100 kg, height less than 150 cm or more than 180 cm, gestational age <37 weeks, active labor, twin or multiple gestation, emergent cesarean section, fetal compromise or known fetal anomaly, contraindication to regional anesthesia due to hemostatic disorder, or refusal to consent to the study or inability to consent due to communication or language barriers. Parturients were additionally excluded due to preexisting or pregnancy-related hypertension, preeclampsia, use of antihypertensive medication, or known cardiovascular or cerebrovascular disease.

Eligible participants were subsequently randomized with the help of a computer-generated sequence of random numbers into either the colloid preloading group or crystalloid co-loading group via the use of the online software QuickCalcs (GraphPad Inc., San Diego, CA, USA). Colloid preloading consisted of 6% hydroxyethylstarch (HES) 130/0.4 administered in the respective group at a volume of 5 mL kg^−1^. Crystalloid co-loading consisted of Ringer’s lactate solution 10 mL kg^−1^ starting simultaneously with the intrathecal injection in the respective group. All group allocations were kept in sealed opaque envelopes, which were sequentially numbered and were disclosed by a separate healthcare practitioner not involved in further patient management upon arrival of the parturient in the operating room to reveal which fluid strategy technique would be followed. The attending anesthesiologist was not blind to the fluid regimen tested, while the parturient and the outcomes assessor were unaware of group allocation.

### 2.2. Anesthetic Procedure

A standardized anesthetic technique as per institutional protocols, including six-hour fasting for solids and two-hour fasting for clear fluids as well as antacid prophylaxis, was provided. On the operating table, parturients were placed in a supine position with a wedge under the right buttock to ensure left lateral uterine tilt. Standard monitoring consisting of three-lead electrocardiogram, non-invasive blood pressure, and continuous pulse oximetry was applied. After a brief resting period, baseline hemodynamic recordings were taken, while baseline blood pressure was recorded as the average of three successive readings taken two minutes apart that did not differ more than 10% among them. After noting baseline hemodynamic data, an indwelling intravenous catheter was inserted in a large forearm vein, and metoclopramide 10 mg was administered as antiemetic prophylaxis. Followingly, in the colloid preload group, 5 mL kg^−1^ of HES was rapidly infused before the performance of the regional technique. In both groups, a second indwelling intravenous catheter was also placed in an upper limb vein and was connected through a three-way stopcock to a 50 mL infusion syringe placed in an electronic infusion pump at stand-by mode via low-volume tubing. This infusion syringe contained norepinephrine that had been prepared by dilution in 5% dextrose solution so that an infusion rate of 30 mL h^−1^ would correspond to 4 mcg min^−1^ of norepinephrine (8 mcg mL^−1^). After colloid administration in the colloid preload group, no additional fluids were administered apart from those required for maintenance of vein patency at a rate of 3 mL kg^−1^ h^−1^ via the second intravenous catheter. In the crystalloid co-load group, maintenance fluid of lactated Ringer’s solution was also initiated at 3 mL kg^−1^ h^−1^ via the second intravenous catheter awaiting the performance of the regional technique.

Following the above-described procedure, parturients were placed in the left lateral decubitus position, and a combined spinal epidural was performed using the needle-through-needle technique at the estimated L_3–4_ or L_4–5_ vertebral interspace as per institutional protocol. An 18-gauge Tuohy epidural needle was used to identify the epidural space using the midline approach and the loss of resistance to saline method after skin disinfection and local skin infiltration with lidocaine 2%. Upon identification of the epidural space, a 27-gauge Whitacre spinal needle with a pencil-point tip was threaded through the Tuohy needle into the subarachnoid space. After confirmation of entry into the subarachnoid space via free cerebrospinal fluid (CSF) flow, the subarachnoid solution consisting of isobaric ropivacaine 0.75% 1.8 mL plus fentanyl 10 mcg was administered. The spinal needle was subsequently withdrawn, and an epidural catheter was threaded into the epidural space via the Tuohy needle. No parturient was administered any local anesthetic through the epidural catheter, which was gently aspirated and checked for the presence of CSF before being securely fixed. The allocated crystalloid co-load of 10 mL kg^−1^ was commenced at the time of initiation of spinal anesthesia in the respective group via the first intravenous catheter inserted and was rapidly infused using a pressurized inflatable bag adjusted to 250 mmHg. In both study groups, the electronic infusion pump syringe containing the norepinephrine solution was initiated at a rate of 30 mL h^−1^ as soon as CSF was visible at the spinal needle hub, just before the administration of the intrathecal medication.

Afterwards, parturients were placed in the level zero supine position, with the wedge under the right buttock as described beforehand, to ensure continued left uterine displacement and to prevent aortocaval compression. Following the completion of the study fluid infusion, maintenance fluid of lactated Ringer’s solution at 3 mL kg^−1^ h^−1^ was continued in the crystalloid co-load group, as in the other group. Sensory level was checked bilaterally by loss of painful pinprick sensation, and only when this level had reached the T_4_ dermatome was the operation allowed to start. If the block was unsuccessful, the parturient was excluded from the study, and the random code was allocated to the next eligible parturient. Administration of the norepinephrine infusion was discontinued five minutes after delivery, and afterwards, the attending anesthesiologist continued to treat hypotension, hypertension, and bradycardia accordingly. Immediately after the delivery of the fetus, oxytocin was administered as a bolus of 3 UI, followed by a slow infusion of 17 UI. No supplementary oxygen was routinely administered unless required as guided by pulse oximetry readings below 95%, in which case 3 L min^−1^ of oxygen was administered via nasal prongs.

### 2.3. Measurements

Hemodynamic parameters included systolic arterial pressure (SAP), diastolic arterial pressure (DAP), mean arterial pressure (MAP), and heart rate (HR) and were measured at specific timepoints during the operation: at baseline (T_0_), at the initiation of the norepinephrine solution (T_1_), when the parturient was positioned supine (T_2_), when the sensory block reached the T4 dermatome (T_3_), when the operation started (T_4_), at fetal delivery (T_5_), five minutes after oxytocin administration (T_6_), and at the end of the operation (T_7_).

Hypotension was defined as SAP <80% of baseline value, while hypertension was defined as SAP >120% of baseline value. Bradycardia was defined as HR <60 beats per minute (bpm). Throughout the operation, a predetermined phenylephrine bolus of 50 mcg was administered in case of hypotension in combination with HR >80 bpm, while an ephedrine bolus of 5 mg was administered when hypotension occurred in combination with HR <80 bpm. Hypertensive episodes (SAP > 120% of baseline) were treated by halving the norepinephrine infusion to 15 mL/h, while when SAP increased above 130% of baseline, the infusion was discontinued. The norepinephrine infusion was maintained at the predetermined rate of 30 mL h^−1^ for a range of SAP within 80–120% of baseline. In case of bradycardia (HR < 60 bpm) in combination with normotension (SAP within 80–120% of baseline) or hypertension, then norepinephrine infusion was discontinued without the administration of atropine. Atropine at a dose of 0.6 mg was administered when HR fell below 55 bpm in combination with hypotension (SAP value < 80% of baseline) or in case of HR <50 bpm irrespective of hypotension.

### 2.4. Study Outcomes

The primary outcome of the study was the incidence of maternal hypotension from the time of the subarachnoid injection until delivery (SAP < 80% of baseline). Secondary outcomes were hemodynamic measurements at the specific timepoints describe above; the incidence of severe maternal hypotension (SAP < 80 mmHg); the number of hypotensive episodes requiring treatment; the incidence of reactive hypertension or bradycardia; any intraoperative requirements for bolus administration of ephedrine, phenylephrine, or atropine; and the total dosage administered. The time from the induction of anesthesia to the first hypotensive episode, any requirement for modification, or cessation of the norepinephrine infusion due to reactive hypertension or bradycardia as well as any incidence of nausea, vomiting, dizziness, or any other side effects in the parturient were also noted and recorded.

Other parameters evaluated were the total volume of fluids and procedure-related intervals, such as duration of operation (min), time from start of subarachnoid infusion to T4 block (min), intrathecal injection to delivery interval (min), skin-incision-to-delivery interval (min) and uterine-incision-to-delivery interval (sec), birth weight of the neonate, and umbilical artery blood gases taken by a double-clamped segment of the umbilical cord (pH, PO_2_, PCO_2_, glucose, lactate, and base excess). Apgar scores were recorded by the attending pediatrician, while any incidence of umbilical artery pH < 7.2 and Apgar score < 7 at 1 min or <9 at 5 min was also noted.

### 2.5. Statistical Analysis

Sample size calculation was based on the primary outcome of maternal hypotension and the information provided by two previous studies, which reported a 40% incidence of hypotension in parturients receiving a norepinephrine infusion of 0.05 mcg kg^−1^ min^−1^ (which is equivalent to the one used in our study) in combination with crystalloid co-loading [20,21]. By assuming that a 70% relative decrease in the incidence of hypotension with the use of norepinephrine in combination with colloid preloading would be clinically meaningful and by applying the Yate’s correction factor, we estimated that a sample size of 45 parturients in each group would achieve a power of 0.80 and an alpha error of 0.05. We aimed for six more parturients per group to allow for subject dropouts and exclusions. The Kolmogorov–Smirnoff test was used to test the normality of distribution of investigated parameters. Comparison of numeric data between the two groups was performed with the unpaired *t*-test or the Mann–Whitney U-test for independent samples depending on whether the variables followed the normal or non-normal distribution. The chi-square test or Fisher’s exact test, as appropriate, were used for comparisons of categorical data. The time from the induction of anesthesia to the first episode of hypotension was analyzed using Kaplan–Meier survival analysis, and differences between the two groups were computed by the log-rank test. Parturients who delivered without any incidence of hypotension were considered “censored”. Serial hemodynamic data (SAP, DAP, MAP, and HR) were analyzed and compared between the two groups with a two-step summary measures technique [22]. Taking into consideration the fact that there was variation in surgical times among parturients, the comparison of mean values at each particular timepoint was not deemed appropriate. In contrast, the area under the curve for values plotted against time was calculated for each parturient, which was then divided by the number of recording points to provide one standardized value. Τhe unpaired *t*-test or Mann–Whitney U-test, as appropriate, was then used to compare standardized hemodynamic data between the two groups. For the variation of hemodynamic data over time within each group, repeated measures analysis of variance (ANOVA) was used (with Bonferroni adjustment). Results are expressed as mean (SD) or as median and interquartile range (IQR), depending on normality of distributions and as absolute numbers (frequency) for categorical variables. Relative risks of binary variables and 95% confidence intervals (CI) of the relative risk were also calculated. A value of *p* < 0.05 was considered as statistically significant. Data were analyzed with the SigmaPlot version 13 for Windows statistical software (Systat Software, Inc., San Jose, CA, USA).

## 3. Results

**Figure 1:**
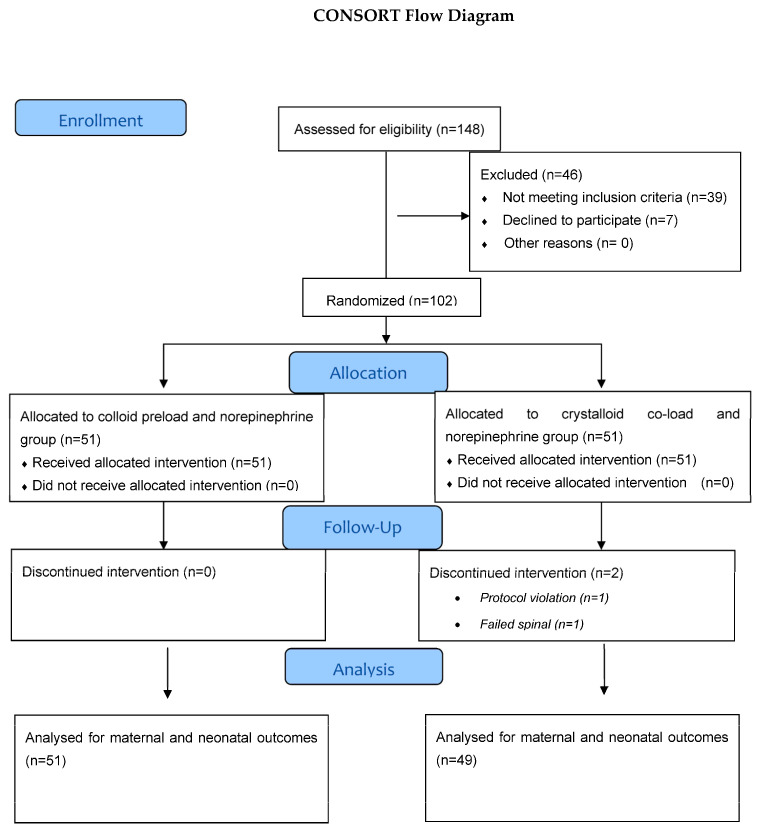
Consolidated Standards of Reporting Trials (CONSORT) chart detailing parturient recruitment and flowing.

Among 148 parturients scheduled for elective cesarean section who were assessed for eligibility, 102 parturients were recruited in the study: colloid preload group (*n* = 51) and crystalloid co-load group (*n* = 51). Data from all parturients were analyzed as per intention-to-treat analysis. In the crystalloid co-load group, two patients were excluded from further analysis due to failed spinal and protocol violation. Only patients who were randomized and received the allocated intervention were included in the final analysis. Therefore, data analysis was performed on 51 parturients in the colloid preload group and 49 parturients in the crystalloid co-load group. The flow chart of the study with parturient recruitment, allocation, follow-up, and analysis is presented in Figure 1.

**Figure 2:**
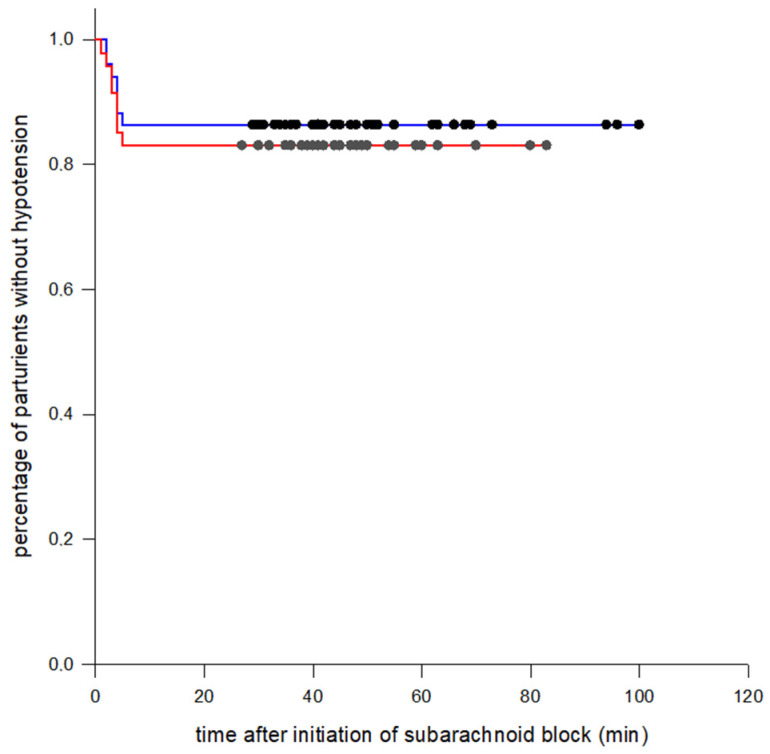
Time to onset of hypotension demonstrating no significant differences between the two groups (colloid preload and norepinephrine, blue line; crystalloid co-load and norepinephrine, red line) (Kaplan–Meier method, log-rank statistic *p* = 0.642). The y-axis depicts the percentage of parturients without hypotension, while the x-axis depicts the time after subarachnoid block (min). Censored parturients are indicated by the black and grey dot tick marks.

Parturient demographic characteristics, surgical times, and baseline hemodynamic parameters were similar between the two groups (Table 1). There was no requirement for any supplementary epidural medication for intraoperative pain in any parturient. Regarding the primary outcome of the study, there was not any significant difference in the incidence of maternal hypotension between the colloid preload and the crystalloid co-load group (13.7% vs. 16.3%; relative risk 0.841, 95% CI 0.330 to 2.143, *p* = 0.933) (Table 2). Similarly, no significant difference in the incidence of severe maternal hypotension was demonstrated between the two groups (0% vs. 4%; relative risk 0.000, 95% CI 0.000 to --, *p* = 0.238). The median number of hypotensive episodes that required treatment were 0 (0–3) and 0 (0–2), *p* = 0.807, in the colloid preload and crystalloid co-load group, respectively. Similarly, the median (range) ephedrine dose was 0 (0–15) mg in the colloid preload group and 0 (0–10) mg in the crystalloid co-load group (*p* = 0.807). There was no requirement for phenylephrine administration in either group. The incidence of bradycardia was low and comparable between the two groups (1.9% vs. 2%; relative risk 0.961, 95% CI 0.061 to 14.938, *p* = 1.0). There was no requirement for atropine administration in either group. The incidence of reactive hypertension was not significantly different between the two groups either (11.7% in the colloid preload group vs. 8.1% in the crystalloid co-load group; relative risk 1.441, 95% CI 0.433 to 4.799, *p* = 0.741). There was no difference in the requirement for modification in the norepinephrine infusion rate due to bradycardia or reactive hypertension between the two groups (13.7% vs. 10.2%; relative risk 1.345, 95% CI 0.457 to 3.955, *p* = 0.815). No parturient complained about nausea, emesis, or dizziness. As expected, the crystalloid co-load group received a greater volume of crystalloid solution as compared to the former group (*p* < 0.001) (Table 2). The time to onset of hypotension after subarachnoid block placement was not significantly different between the two groups (log-rank test, *p*-statistic 0.642) (Figure 2).

**Figure 3:**
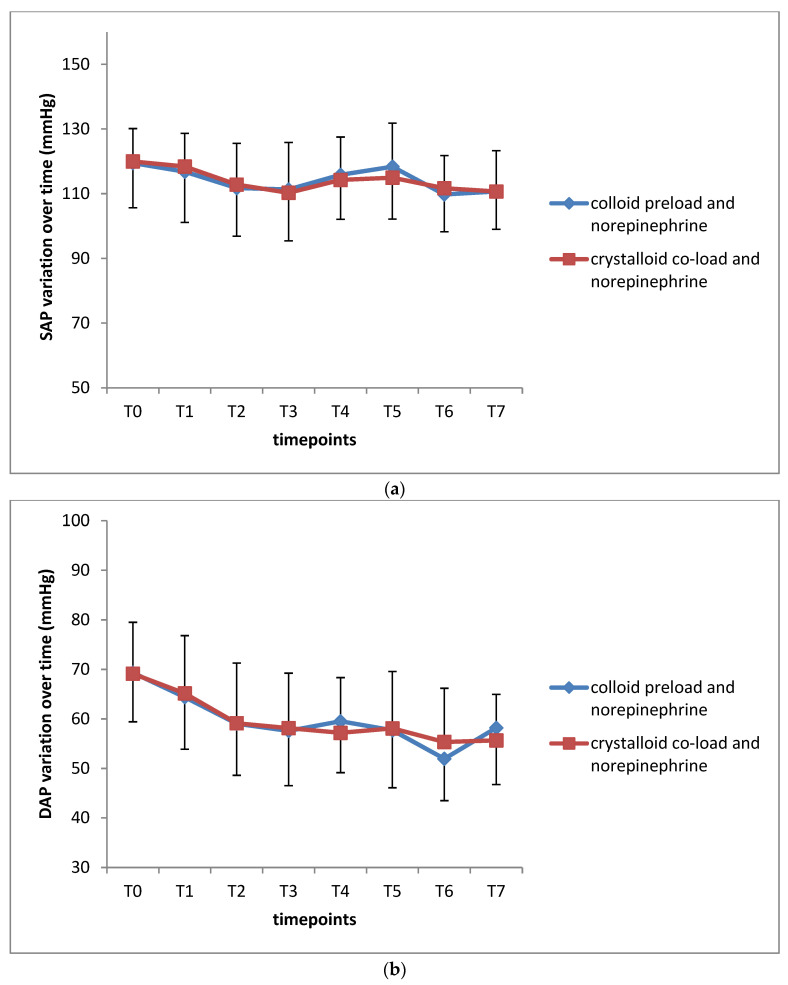

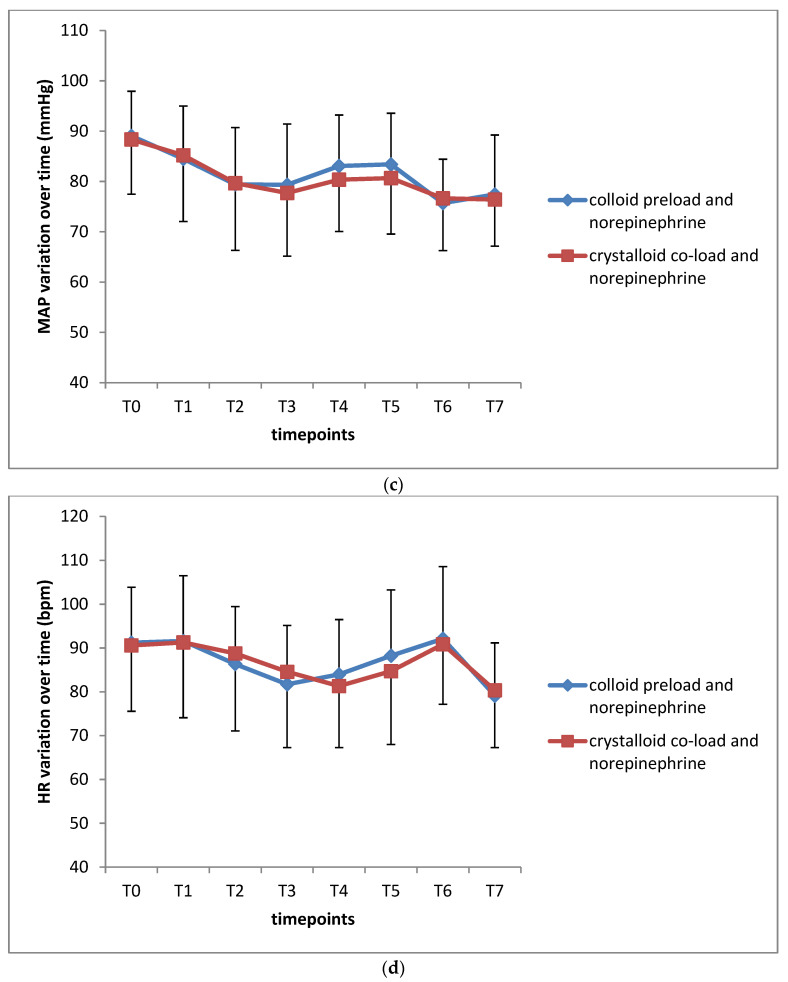
(**a**) Serial changes in systolic arterial pressure (SAP) over time for the group colloid preload and norepinephrine versus the group crystalloid co-load and norepinephrine. Data are depicted as mean (SD). Comparison between the two groups did not demonstrate a significant difference between the two groups (standardized SAP in the first group 114.2 (9.2) versus 113.9 (11.5) in the second group, *p* = 0.890). There were significant time effects (*p* < 0.001) within each group for SAP. Timepoints: baseline (T_0_), start of “study infusion” medication (T_1_), parturient at supine position (T_2_), sympathetic block at T4 dermatome (T_3_), start of operation (T_4_), delivery of fetus (T_5_), 5 min after oxytocin administration (T_6_), and end of operation (T_7_). (**b**) Serial changes in diastolic arterial pressure (DAP) over time for the group colloid preload and norepinephrine versus the group crystalloid co-load and norepinephrine. Data are depicted as mean (SD). Comparison between the two groups did not demonstrate a significant difference between the two groups (standardized DAP in the first group 59.7 (6.8) versus 59.9 (8.2) in the second group, *p* = 0.867). There were significant time effects (*p* < 0.001) within each group for DAP. Timepoints: baseline (T_0_), start of “study infusion” medication (T_1_), parturient at supine position (T_2_), sympathetic block at T4 dermatome (T_3_), start of operation (T_4_), delivery of fetus (T_5_), 5 min after oxytocin administration (T_6_), and end of operation (T_7_). (**c**) Serial changes in mean arterial pressure (MAP) over time for the group colloid preload and norepinephrine versus the group crystalloid co-load and norepinephrine. Data are depicted as mean (SD). Comparison between the two groups did not demonstrate a significant difference between the two groups (standardized MAP in the first group 81.6 (7.7) versus 80.6 (8.6) in the second group, *p* = 0.570). There were significant time effects (*p* < 0.001) within each group for MAP. Timepoints: baseline (T_0_), start of “study infusion” medication (T_1_), parturient at supine position (T_2_), sympathetic block at T4 dermatome (T_3_), start of operation (T_4_), delivery of fetus (T_5_), 5 min after oxytocin administration (T_6_), and end of operation (T_7_). (**d**) Serial changes in heart rate (HR) over time for the group colloid preload and norepinephrine versus the group crystalloid co-load and norepinephrine. Data are depicted as mean (SD). Comparison between the two groups did not demonstrate a significant difference between the two groups (standardized HR in the first group 86.8 (10.7) versus 86.6 (12.4) in the second group, *p* = 0.950). There were significant time effects (*p* < 0.001) within each group for HR. Timepoints: baseline (T_0_), start of “study infusion” medication (T_1_), parturient at supine position (T_2_), sympathetic block at T4 dermatome (T_3_), start of operation (T_4_), delivery of fetus (T_5_), 5 min after oxytocin administration (T_6_), and end of operation (T_7_).

No significant intergroup differences were observed in standardized SAP, DAP, MAP, and HR (*p* = 0.890 for SAP, 0.867 for DAP, 0.570 for MAP, and 0.950 for HR) (Table 2). Serial changes of hemodynamic parameters are depicted in Figure 3a–d. No differences over time between the two groups were demonstrated. There were significant time effects (*p* < 0.001) within each group for all hemodynamic data analyzed.

Neonatal outcomes are presented in Table 3. No differences in neonatal blood gases were demonstrated between the two groups. There was a marginal difference in neonatal glucose levels, which, however, is clinically unimportant. Similarly, birth weight and Apgar scores at 1 and 5 min postdelivery presented no difference between the two groups, while there was not any need for manual assistance of ventilation in any neonate. One neonate in each group had pH less than 7.2, while all Apgar scores were ≥7 at one minute and ≥9 at five minutes postdelivery.

## 4. Discussion

According to the results of this randomized controlled trial, the incidence of hypotension during elective cesarean section is low and comparable when a norepinephrine infusion is used in combination with either colloid preload or crystalloid co-load. All other additional outcomes including the incidence of bradycardia, the requirement for rescue vasopressor boluses, the requirement for modification in the vasoactive infusion rate resulting from episodes of bradycardia or reactive hypertension, and maternal side effects were also similar between the two fluid-loading regimens. Furthermore, there was no difference in neonatal outcome and metabolic profile of the neonate, as expressed by the acid–base status.

Spinal anesthesia for cesarean section can be very frequently complicated by hypotension. In the past, it was thought that hypotension in the context of regional anesthesia for cesarean section was due to the reduction of venous return and the accompanying fall of ventricular filling and cardiac output. Therefore, early research to prevent or minimize spinal hypotension focused primarily on techniques aiming at increasing blood volume and venous return, such as fluid loading, positioning to minimize aorto-caval compression, elevation of the lower limbs, and leg wrapping. However, these strategies have proved ineffective in restoring maternal blood pressure [23]. More recent studies suggest that spinal hypotension is mainly driven by a decrease in sympathetic tone in the arterial system accompanied by venodilation, resulting in a decrease in stressed blood volume and an increase in unstressed volume [24]. Actually, it has been demonstrated by studies using minimally invasive cardiac output monitoring that a marked reduction in systemic vascular resistance and a modest increase in cardiac output occur after induction of spinal anesthesia [2,25]. A recent network meta-analysis aiming at determining the relative efficacy of methods to prevent hypotension during elective cesarean section corroborated the fact that maternal hypotension, nausea, and vomiting are less common with the use of vasopressors than with measures aiming at increasing central blood volume, such as fluid loading and leg compression [14]. Therefore, nowadays, vasopressors are considered the most appropriate method for preventing and treating spinal hypotension during cesarean section and are considered the mainstream of management since they address the iatrogenic sympathectomy and the primary physiologic derangement induced by the sympathetic block, that is, arterial vasodilation and reduced systemic vascular resistance. Additionally, by restoring and maintaining vascular tone in the capacitance side of the circulation (venous and splanchnic vessels), vasopressors also maintain venous return and cardiac filling, enhancing unstressed-to-stressed blood volume conversion [3,14,24,26].

Our institution promotes shortened starvation times and proactive fluid administration prior to surgery. However, this has not been shown to really prevent spinal-anesthesia-induced hypotension [27]. Therefore, in our study, we used a continuous infusion of norepinephrine in combination with fluid loading for the prevention of maternal hypotension. We opted for a continuous infusion regimen of norepinephrine as opposed to rescue bolus administration. Continuous vasopressor infusions are favored in the obstetric setting since they are more effective in preventing hypotension rather than treating it, they allow tighter pressure control, they reduce hemodynamic fluctuations and maternal nausea and vomiting, and they limit clinician workload by requiring minimal interventions by the anesthesiologist [28,29]. Additionally, we used a standardized infusion of norepinephrine, not basing the dosing regimen on body weight. The concept of fixed-rate vasoactive infusion is not new in the obstetric literature and has been explored for both phenylephrine and norepinephrine infusions [4,30]. In fact, Ngan Kee has noted that although adjusting dose according to weight seems to make pharmacological sense, many clinicians may prefer to use non-weight-adjusted infusions for simplicity [3]. Additionally, in a study comparing a fixed-rate regimen of phenylephrine versus a variable-rate regimen for prevention of maternal hypotension, Hasanin et al. demonstrated that the former is associated with a lower number of physician interventions and unnecessary calculations [31].

We selected a fixed-dose regimen of 4 mcg min^−1^ for norepinephrine based on studies exploring similar dosing schemes. Specifically, manual titration of a norepinephrine infusion within a range of 0–5 mcg min^−1^ without relying on body weight for calculations was shown to be effective in maintaining normotension in parturients according to a dose-finding trial by Ngan Kee et al. [30]. In another dose-finding study in which three groups of parturients receiving norepinephrine with starting infusion rates of 0.025, 0.05, and 0.075 mcg kg^−1^ min^−1^ were compared, it was shown that the two higher rates were more effective in decreasing the frequency of maternal hypotension [32]. However, the authors suggested that the higher dose infusion of 0.075 mcg kg^−1^ min^−1^ can lead to a higher incidence of hypertension, offering no additional advantage in comparison to the 0.05 mcg kg^−1^ min^−1^ dose; therefore, they recommended 0.05 mcg kg^−1^ min^−1^ as the optimal dosing scheme. Finally, in a recent dose-finding study, ED_50_ for continuous norepinephrine infusion was calculated as 0.029 mcg kg^−1^ min^−1^, while ED_80_ was calculated as 0.068 mcg kg^−1^ min^−1^ [20]. Based on these dosing schemes, we opted for a fixed dose of 4 mcg min^−1^, which approximates the recommended infusion regimens mentioned above for parturients of usual weights encountered in the everyday anesthetic practice. On top of this, simplified non-weight-based infusion regimens have additional advantages, as they are easier to prepare and administer since, with their use, unnecessary calculations and adjustments can be avoided.

We specifically investigated a comparison scarcely examined in the literature, that is, the comparison between colloid preload and crystalloid co-load. Crystalloids as well as colloids have been widely used in both preloading and co-loading strategies in the obstetric anesthesia context [15]. Advantages of crystalloids include the lack of untoward side-effects in combination with their low cost. On the other hand, their efficacy in intravascular space filling is low since only 20% of any crystalloid solution remains in the circulation due to rapid distribution to the interstitium, which limits the effective augmentation of intravascular volume [33,34]. On the other hand, colloid administration can be complicated by untoward effects such as anaphylactoid reactions and renal function compromise. When crystalloid solutions are compared to colloids in the management of maternal hypotension, the latter have been shown to be superior at least in the context of preloading [35]. In the setting of co-loading, existing evidence suggests equal effectiveness of colloids and crystalloids in the prevention of hypotension [36]. In light of this, the meta analyses performed have also suggested that colloid administration acts favorably in the occurrence of hypotension and the requirement of vasoconstrictor administration, without, however, any advantage in neonatal outcomes [13,14].

We did not demonstrate any difference in maternal and neonatal outcomes with the two fluid-loading regimes we used: timed either before or after establishment of the spinal block. In fact, the ideal timing of fluid administration during cesarean section continues to be a debatable issue. The rationale behind preloading is the filling of intravascular space before local-anesthetic-induced vasodilation leads to relative hypovolemia. However, rapid crystalloid administration as a preload causes atrial stretch and leads to the release of atrial natriuretic peptide (ANP), which in turn induces peripheral vasodilation and diuresis, thus attenuating the favorable effect of fluid loading and reducing sensitivity to vasoconstrictors administered during cesarean section [37,38]. Additionally, iatrogenic hypervolemia associated with rapid crystalloid administration has been implicated in the occurrence of marked endothelial injury in experimental models [39]. On the other hand, co-loading (fluid administration during or after subarachnoid anesthesia initiation) aims at filling the central compartment since sympathetic blockade leads to fluid shift to the peripheral compartment of lower extremities and splanchnic network. Thus, co-loading exerts its maximal effect as the spinal block occurs since it fills the intravascular space as it expands secondary to spinal anesthesia-induced sympathectomy, while redistribution into the interstitial compartment decreases due to the drop in hydrostatic pressure accompanying spinal-induced vasodilation [40]. Furthermore, during co-loading, fluid is infused as the intrathecal injection of local anesthetic causes vasodilation, so it is expected to cause less atrial stretch and consequently less ANP secretion than if administered before spinal anesthesia. In other words, with preloading, fluid is administered when a virtual physiological state between unstressed and stressed blood volume exists; therefore, the body attempts to eliminate this extra fluid via movement into the extravascular space and excretion by the kidneys. In contrast, with co-loading, the fluid is administered exactly when the steady state is being disturbed at the time of the spinal-induced vasodilation, and therefore, the fluid load can be used to replace the decreased stressed volume, and thus, fluid redistribution and excretion is limited [24]. In this context, preloading of crystalloids has proven to be a totally ineffective strategy as compared to co-loading of crystalloids in the prevention of maternal hypotension since infusing fluids after the block maximizes the amount of volume in the functional compartment [13,14,16]. Regarding the optimal time of colloid administration, available evidence suggests that preloading may be equally effective to co-loading regarding the incidence of hypotension and neonatal outcome since colloids reside in the intravascular compartment long enough to be effective during spinal-induced sympathectomy even after having been administered as preload. Therefore, there is not any overt benefit in delaying spinal anesthesia so that fluids can be pre-administered or expediting spinal anesthesia so that fluid administration can follow its performance [41].

In both groups of parturients in our study, marked hemodynamic stability was demonstrated. The incidence of hypotension was low and comparable between the two fluid regimens. In the only study we could identify available in the literature, that by Tawfik et al., where colloid preload was compared to crystalloid co-load, the incidence of hypotension was much higher than in our study (52.4% and 42.2 % in the two groups, respectively) [18]. However, in the Tawfik study, no vasopressor infusion was simultaneously administered, which might account for the higher incidence of hypotension in comparison to our study.

The incidence of bradycardia was also low in both groups, an effect mainly attributed to the favorable action of the norepinephrine solution, whose β-adrenergic receptor activity counterbalances the reflex slowing of heart rate due to the potent α-adrenergic effect. The decreased incidence of bradycardia with the use of norepinephrine is in accordance with other published studies and has also been corroborated in reviews and meta analyses [5,6,7,8]. Better maintenance of cardiac output can also be speculated with dosing regimens based on norepinephrine since heart rate is a satisfactory surrogate marker of cardiac output, and a good correlation between heart rate and cardiac output has already been demonstrated [28,42]. Neonatal outcomes were also unaffected by the technique of fluid administration. This further emphasizes the importance of early recognition and timely and effective treatment of maternal hypotension as the main factor in ensuring optimal outcome rather than relying on the type of fluid used. We should, however, note the large placental reserve in healthy pregnancy and the fact that our study was not powered to the occurrence of specific neonatal outcomes.

Our study has a few limitations such as the aforementioned lack of direct measurement of cardiac output and the enrollment of only healthy parturients without cardiac derangement, coexisting maternal disease, fetal compromise, abnormal BMI, or emergent cesarean delivery. It would be of interest to investigate the favorable effect of norepinephrine in combination with either regime of fluid loading on cardiac output or to ascertain whether our findings could be extrapolated to non-elective cesarean sections or to cases with compromised cardiac function. An additional limitation is the use of a fixed-rate infusion of norepinephrine since it could be argued that variable weight-based rate infusion regimes tightly control blood pressure within a narrower individualized range to ensure optimal hemodynamic control. Finally, the attending anesthesiologist was not blind to the fluid-loading technique used; however, the assessor who analyzed parturient data was unaware of group allocation. The strength of our study lies in the fact that we performed a comparison never attempted before in the literature.

The mainstay of prevention of maternal hypotension seems to be a vasoactive substance such as norepinephrine in combination with a fluid maintenance strategy. Purported advantages of norepinephrine include rapid onset and short duration of action (enabling accurate titration), reduced cost, and wide availability in most countries. Fixed-rate infusions are also a popular solution for most institutions since simplicity of preparations enables easy use even in low-resource settings. It appears that the co-administration of a continuous infusion of norepinephrine with either colloid preload or crystalloid co-load ensures optimal hemodynamic stability. Additionally, since it has been shown in dose–response studies that increasing infusion rates of norepinephrine decrease the incidence of hypotension [20,21], it could be hypothesized that increased infusion rates could result in reduced requirements for fluid loading. This interesting hypothesis could be tested in future studies. Finally, our results are particularly relevant in view of the recent warning by the U.S. Food and Drug Administration and the European Medicines Agency about regulatory restrictions in the use of colloids in critically ill patients due to fear of anaphylactoid reactions, kidney injury, and coagulopathy [43]. Although the restrictions apply to critically ill patients, and it remains unclear whether the same risks exist in the perioperative setting (including cesarean delivery), it appears that since no clear benefits of colloids were found, crystalloid co-loading is a viable and less costly alternative strategy in this context, obviating the need to delay anesthesia for fluid administration beforehand. In fact, in a recent network meta-analysis, Rijs recommended crystalloid co-load as the most appropriate fluid regimen [10].

In conclusion, we found no benefit from administration of colloid preload compared with crystalloid co-load for preventing hypotension in parturients receiving a prophylactic norepinephrine infusion during cesarean delivery. It seems that the norepinephrine infusion can be combined with either colloid preload or crystalloid co-load to achieve the best efficacy, and it remains to be seen whether the proposed combinations could also be safely applied in case of parturients with cardiac comorbidities, uteroplacental compromise, or emergent cesarean sections. Therefore, as long as a continuous infusion of norepinephrine is used, time and type of co-administered fluids do not seem to matter.

## Figures and Tables

**Table 1:** Parturient Demographic Characteristics, Surgical Times, and Baseline Hemodynamics.

Characteristics	Colloid Preload and Norepinephrine (N = 51)	Crystalloid Co-Load and Norepinephrine (N = 49)	*p*-Value, Group Comparison
Maternal age (years), mean (SD)	31.2 (6.6)	33.0 (5.4)	0.149
Weight at term (kg), mean (SD)	65.5 (11.2)	67.4 (10.7)	0.397
Height (cm), mean (SD)	164.1 (5.8)	164.9 (5.5)	0.507
Gestational age (weeks), median (IQR]	38 (38–39)	38 (38–39)	0.880
Parity (n), median (IQR)	2 (1–2)	1 (1–2)	0.257
Start of subarachnoid infusion-delivery time (min), median (IQR)	22 (20–26)	21 (18–23.25)	0.131
Skin incision-delivery time (min), median (IQR)	7 (5–8)	7 (6–9)	0.262
Uterine incision-delivery time (s), median (IQR)	60 (60–90)	90 (60–120)	0.086
Duration of operation (min), median (IQR)	44 (34–54.25)	44 (40–53)	0.552
Baseline SAP (mmHg), mean (SD)	119.4 (10.6)	119.9 (14.3)	0.846
Baseline DAP (mmHg), mean (SD)	69.3 (9.9)	69.1 (10.3)	0.925
Baseline MAP (mmHg), mean (SD)	89.0 (8.8)	88.3 (10.8)	0.720
Baseline HR (bpm), mean (SD)	91.2 (12.6)	90.5 (15.0)	0.822

abbreviations: SD, standard deviation; IQR, interquartile range; n, number; N, total group number; SAP, systolic arterial pressure; DAP, diastolic arterial pressure; MAP, mean arterial pressure; HR, heart rate.

**Table 2:** Maternal outcomes.

Variables	Colloid Preload and Norepinephrine (N = 51)	Crystalloid Co-Load and Norepinephrine (N = 49)	Relative Risk or Mean Difference (95% CI)	*p*-Value, Group Comparison
Incidence of hypotension, n/N (%) ^a,b^	7/51 (13.7%)	8/49 (16.3%)	0.84 (0.33 to 2.14)	0.933
Incidence of severe hypotension, n/N (%) ^c^	0/51 (0.0%)	2/49 (4.0%)	0.00 (0.00 to --)	0.238
Number of hypotensive episodes requiring treatment, median (IQR (range))	0 (0–0 (0–3))	0 (0–0 (0–2)		0.807
Total dose of ephedrine (mg), median (IQR (range))	0 (0–0 (0–15))	0 (0–0 (0–10))		0.807
Treatment of hypotension with phenylephrine, n/N (%)	0/51 (0.0%)	0/49 (0.0%)		-
Total volume of crystalloids administered, mL, median (IQR (range))	150 (150–250 (100–500))	1100 (1100–1200 (1000–1500)) *		**<0.001**
Total volume of colloids administered, mL, median (IQR (range))	400 (350–450 (250–500))	0 (0–0 (0–0)) *		**<0.001**
Incidence of bradycardia, n/N (%)	1/51 (1.9%)	1/49 (2.0%)	0.96 (0.06 to 14.93)	1.0
Incidence of reactive hypertension, n/N (%)	6/51(11.7%)	4/49 (8.1%)	1.44 (0.43 to 4.79)	0.741
Requirement for modification in infusion rate, n/N (%)	7/51 (13.7%)	5/49 (10.2%)	1.34 (0.45 to 3.95)	0.815
Standardized SAP over time (mmHg), mean (SD)	114.2 (9.2)	113.9 (11.5)		0.890
Standardized DAP over time (mmHg), mean (SD)	59.7 (6.8)	59.9 (8.2)		0.867
Standardized MAP over time (mmHg), mean (SD)	81.6 (7.7)	80.6 (8.6)		0.570
Standardized HR over time (bpm), mean (SD)	86.8 (10.7)	86.6 (12.4)		0.950

* significant difference between groups. abbreviations: n, number; N, total group number; CI, confidence interval; IQR, interquartile range; SAP, systolic arterial pressure; SD, standard deviation; DAP, diastolic arterial pressure; MAP, mean arterial pressure; HR, heart rate; bold is for significant differences. ^a^ Primary outcome. ^b^ Defined as systolic blood pressure <80% of baseline. ^c^ Defined as systolic blood pressure 80 mmHg.

**Table 3:** Neonatal outcomes.

Variables	Colloid Preload and Norepinephrine (N = 51)	Crystalloid Co-Load and Norepinephrine (N = 49)	*p*-Value, Group Comparison
UA pH, median (IQR)	7.35 (7.32–7.36)	7.35 (7.32–7.36)	0.704
Incidence of UA pH < 7.2, n/N (%)	1 (1.9%)	1 (2.0%)	1.000
UA PO2, mmHg, median (IQR)	20 (16.25–25)	21 (16.5–26)	0.614
UA PCO2, mmHg, median (IQR)	44 (40–48.75)	44 (41–47)	0.911
UA HCO3, meq/L, median (IQR)	23.7 (21.7–25.7)	22.15 (21.05–24.55)	0.096
UA Base excess, meq/L, median (IQR)	0.1 (−2.35 to 1.05)	−1.05 (−2.8 to 0.7)	0.254
UA Lactate, mmol/L, median (IQR)	1.7 (1.4–2.05)	1.7 (1.5–2.2)	0.419
UA Glucose, mg/dL, median (IQR)	70 (65–77)	68 (60.5–73.5) *	**0.042**
Apgar score			
At 1 min median (IQR)	9 (8–9)	9 (8–9)	0.727
At 5 min median (IQR)	9 (9–9)	9 (9–9)	0.192
Incidence of 1-min Apgar score < 7, n/N (%)	0 (0.0%)	0 (0.0%)	-
Incidence of 5-min Apgar score < 9, n/N (%)	0 (0.0%)	0 (0.0%)	-
Birth weight, g, mean (SD)	3291.0 (415.6)	3308.3 (329.3)	0.827

* significant difference between groups. abbreviations: UA, umbilical artery; PO2, partial pressure of oxygen; PCO2, partial pressure of carbon dioxide; HCO3, bicarbonate; IQR, interquartile range; SD, standard deviation; bold is for significant differences.

## Data Availability

The datasets generated during and/or analyzed during the current study are available from the corresponding author on reasonable request.
